# Inexpensive, Accurate, and Stable Method to Quantitate Blood Alanine Aminotransferase (ALT) Levels

**DOI:** 10.3390/mps5050081

**Published:** 2022-10-14

**Authors:** Phillipp Hartmann, Bernd Schnabl

**Affiliations:** 1 Department of Pediatrics, University of California San Diego, La Jolla, CA 92093-0984, USA; 2 Division of Gastroenterology, Hepatology & Nutrition, Rady Children’s Hospital San Diego, San Diego, CA 92123-5030, USA; 3 Department of Medicine, University of California San Diego, La Jolla, CA 92093-0063, USA; 4 Department of Medicine, VA San Diego Healthcare System, San Diego, CA 92161-0002, USA

**Keywords:** ALT, liver disease, EDTA, hemolysis, triglycerides

## Abstract

Alanine aminotransferase (ALT) levels are frequently determined in serum and plasma samples and are a primary measure to quantitate hepatocellular injury in rodents, humans, and other organisms. An accurate, reliable, and scalable assay is hence of central importance. Here, we describe a methodology that fulfills those requirements, and demonstrates an excellent performance similar to a commercial ALT kit, with a long stable performance over several subsequent runs. Further, anticoagulation of blood samples with ethylenediaminetetraacetic acid (EDTA) or heparin results in similar ALT concentrations with this assay, whereas no anticoagulation significantly increases ALT levels. Mild hemolysis does not significantly increase ALT levels; however, moderate to severe hemolysis does lead to higher ALT levels. The assay provides stable results over a wide range of associated triglyceride concentrations that can be expected in serum and plasma samples from rodents and humans with dyslipidemia. It also performs well in diluted samples with a reduction of ALT levels corresponding to the factor used to dilute the samples. The described ALT reagent is also very affordable, costing less than 1/80 of comparable commercial kits. Based on the characteristics above, this methodology is suitable for a broad spectrum of applications in mice and possibly humans, where ALT concentrations need to be determined.

## 1. Introduction

Alanine aminotransferase (ALT) levels are a primary measure to quantitate liver cell injury in a wide spectrum of liver pathologies, including alcohol-associated liver disease [1,2,3,4], non-alcoholic fatty liver disease/diet-induced steatohepatitis [5,6], as well as cholestatic liver disease and other chronic liver diseases [7,8]. Due to supply chain shortages of commercially available ALT kits during the COVID pandemic, we developed a simple, affordable, scalable methodology with high accuracy and reliability. Various techniques have been applied in commercial kits to quantitate ALT concentrations, including enzyme-linked immunosorbent assays (ELISAs) [9], sodium dodecyl sulfate–polyacrylamide gel electrophoresis (SDS-PAGE) [10], or kinetic assays based on enzymatic reactions [11]. Most frequently, ALT concentrations are determined in plasma or serum samples [9,11], but its concentration can also be quantitated in tissue, in particular liver tissue [10]. The most widely clinically adopted method of quantifying the serum ALT concentration relies on spectrophotometric detection [12], which can occur by measuring pyruvate, a product of the reaction catalyzed by ALT, at 540–570 nm [13,14], or indirectly by measuring the concentration of the substrate of a secondary reaction, reduced nicotinamide adenine dinucleotide (NADH) (as detailed below), at 340 nm [12,15,16], similar to our presented method. Here, we detail a methodology that is based on an enzymatic reaction sequence [11]: ALT catalyzes the reaction L-alanine + alpha-ketoglutarate → pyruvate + L-glutamate, whereas lactate dehydrogenase (LDH) catalyzes the reaction pyruvate + NADH + H^+^→ lactate + NAD^+^ + H_2_O. The activity of ALT is quantitated by determining the oxidation rate of NADH. The assay has an excellent performance similar to commercial ALT kits, but is also easily scalable, inexpensive, and very stable.

## 2. Experimental Design

### 2.1. Materials

1. Tris Base (#BP152-1, Fisher Scientific, Waltham, MA, USA);
2. Alpha-ketoglutarate (#75890-25G, Sigma Aldrich, St. Louis, MO, USA);
3. L-alanine (#A7627-100G, Sigma Aldrich, St. Louis, MO, USA);
4. Ethylenediaminetetraacetic acid (EDTA) (#E177-500ML, VWR, Radnor, PA, USA);
5. LDH (#350-20-1, Lee Bio, Maryland Heights, MO, USA);
6. NADH (#16078, Cayman Chemical, Ann Arbor, MI, USA);
7. Phosphate-buffered saline (PBS) (#BP66150, Fisher Scientific, Waltham, MA, USA);
8. Validate GC3 ALT Linearity Test Kit (#1300sd, LGC Maine Standards/LGC Clinical Diagnostics, Inc., Cumberland Foreside, ME, USA).

### 2.2. Equipment

- 96-well flat-bottom plates (#07-200-656, Corning, Corning, NY, USA);
- 15 mL or 50 mL Falcon tube (#1495949B or #1495949A, Fisher Scientific, Waltham, MA, USA);
- Whatman Puradisc 25 mm 0.2 µM polyethersulfone (PES) membrane filter (#6780-2502, Cytiva, Marlborough, MA, USA);
- Disposable reagent reservoirs (#89108-008, Axygen Scientific, Union City, CA, USA);
- VersaMax^TM^ Absorbance Microplate Reader (#VERSAMAX, Molecular Devices, San Jose, CA, USA);
- SoftMax Pro GxP Software version 5.4 (#SMP54-GXP-10UL, Molecular Devices, San Jose, CA, USA).

### 2.3. Methods for ALT Reagent Formulations, Mouse Studies, Triglyceride Experiments and Statistics

#### 2.3.1. Various ALT Reagent Formulations

Multiple ALT reagents were prepared following the instructions per protocol described under point “3. Procedure” below, with the exception of different NADH concentrations, which were chosen to demonstrate the importance of the NADH concentration for the success of the assay. A “low NADH” reagent was prepared with 8 mg/100 mL = 0.12 mM NADH concentration; an “intermediate NADH” reagent was prepared with 50 mg/100 mL = 0.75 mM NADH concentration; and a “high NADH” reagent was prepared with 100 mg/100 mL = 1.5 mM NADH concentration, which corresponds to the NADH concentration per protocol under “3. Procedure” below.

A commercial ALT kit (ALT Kinetic Assay, #A524-150, Teco Diagnostics, Anaheim, CA, USA) was used to evaluate the performance of the developed ALT reagent. The commercial ALT kit included the buffer reagent “R1” and co-enzyme reagent “R2”, which were mixed 5:1 right before the start of the assay per manufacturer’s instructions, to prepare a reagent containing 500 mM L-alanine, >1200 units/L LDH, 100 mM Tris buffer pH 7.5, 15 mM 2-oxoglutarate (=alpha-ketoglutarate), 0.18 mM NADH, and stabilizers and preservatives.

#### 2.3.2. Blood Samples

Blood was obtained from the inferior vena cava from 14 to 15 6- to 10-week-old wild-type C57BL/6 mice, bred at the University of California San Diego. To elicit hepatocellular injury and hence higher ALT levels, a single dose of ethanol (5 g/kg body weight) was gavaged to the mice 9 h prior to sacrifice and blood draw. Immediately prior to sacrifice, mice were anesthetized with 400 µL sodium chloride with 6% ketamine and 1% xylazine per 20 g bodyweight per intraperitoneal injection. Complete anesthesia was confirmed by cessation of physical movements after stimulation. Mice expired by exsanguination; however, additional cervical dislocation was performed after blood was obtained. For the anticoagulation studies, 100–150 µL of blood was added to a sterile Eppendorf tube without any anticoagulant (“no anticoagulant group” = serum), another 100–150 µL of blood from the same mouse was added to a sterile Eppendorf tube with 10 units of heparin (“heparin group” = heparin-treated plasma), and another ~300 µL of blood also from the same mouse was added to a sterile Eppendorf tube with 5 µL EDTA (“EDTA group” = EDTA-treated plasma).

For the hemolysis experiments, ~300 µL of blood was added to a sterile Eppendorf tube with 5 µL EDTA (“no hemolysis group”). Another 100–150 µL of blood from the same mouse was added to a sterile Eppendorf tube with 5 µL EDTA and the red blood cells were then lysed by using the Mini-BeadBeater-96 (#1001, BioSpec, Bartlesville, OK, USA) for 30 s (“severe hemolysis group”). Two additional groups, the “mild hemolysis group” and “moderate hemolysis group” were prepared by mixing volumes of the “no hemolysis group” and the “severe hemolysis group” in 2:1 and 1:2 mixtures for each mouse sample, respectively.

For the dilution experiments, “EDTA group” plasma samples were diluted with sterile water in a 1:2, 1:5, and 1:10 dilution, respectively. Animals were maintained in a temperature-controlled room (22 degrees C) on a 12:12-h light-dark cycle. Animal breeding and harvesting were approved by the Institutional Animal Care and Use Committee of the University of California San Diego.

#### 2.3.3. Triglyceride Experiments

Varying concentrations with purified triglyceride mixes including 20% glyceryl tridecanoate, 20% glyceryl tridodecanoate, 20% glyceryl trimyristate, 20% glyceryl trioctanoate, and 20% tripalmitin (Lipid standards: triglyceride mixtures, #17811-1AMP, Sigma Aldrich, St. Louis, MO, USA) were prepared. Purified triglycerides mixed with sterile water were added to respective ALT standards to achieve final ALT levels.

#### 2.3.4. Statistics

Results are expressed as mean ± s.e.m. Numbers for biological replicates are *n* = 14–15, unless denoted differently in the figure legends. Two technical replicates were performed for each group. Pearson correlation was employed to calculate correlation coefficients between maximum reaction velocity *V_max_* values and ALT values. Significance was evaluated using one-way paired analysis of variance (ANOVA) or–if there are missing values–mixed-effects analysis with Holm-Šídák’s post hoc test. A *p* value < 0.05 was considered to be statistically significant. Statistical analysis was performed using GraphPad Prism 8.4.0 for Mac.

## 3. Procedure

### 3.1. Preparation of Plasma Sample

- Obtain blood from rodents, humans or other organisms. We recommend adding 5 µL of EDTA per 500 µL blood to prevent coagulation.
- Spin blood at 10,000× *g* for 15 min at 4 degrees Celsius.
- Transfer clear supernatant (=plasma) to a new sterile Eppendorf tube and store at −80 degrees Celsius or use immediately for the ALT assay.

### 3.2. Preparation of ALT Reagent

1. Preparation of 100 mL ALT reagent–with 97 millimolar (mM) Tris Base (stored at room temperature), 13 mM alpha-ketoglutarate (stored at 4 degrees Celsius), 440 mM L-alanine (stored at room temperature), 5 mM EDTA (stored at room temperature), 3200 units/L LDH (stored at −20 degrees Celsius), and 1.5 mM NADH (stored at −20 degrees Celsius):
2. Add 1.175 g Tris Base to a glass beaker.
3. Fill up to ~50 mL with sterile water.
4. Start stirring with a magnet stir bar in the beaker.
5. Add 190.0 mg alpha-ketoglutarate.
6. Add 3.92 g L-alanine.
7. Add 1 mL of 0.5 M solution of the preservative EDTA.
8. Dissolve 1000 units of LDH in a vial with 1 mL PBS, and add 320 µL of LDH solution (=320 units) to ALT reagent (store remaining LDH in vial at −20 degrees Celsius).
9. Continue stirring with the magnet stir bar, until all particles are dissolved in the ALT reagent solution.
10. Adjust pH to 7.80 at room temperature.
11. Fill up to final volume of 100 mL with sterile water, transfer this prepared ALT reagent to a sterile bottle, and **PAUSE STEP** store at 4 degrees Celsius protected from a light source (e.g., wrap bottle with aluminum foil) until use.
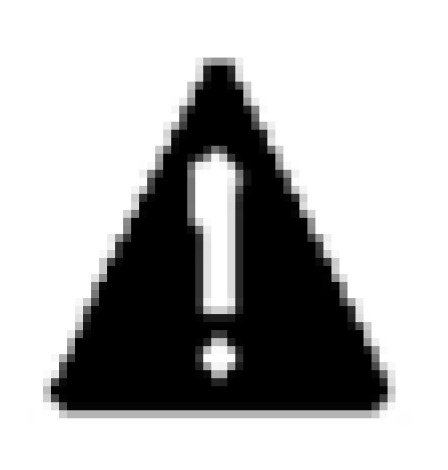
**CRITICAL STEP**Prior to use, obtain the required volume and add the same amount of NADH in mg as the required volume in mL (e.g., add 10 mg NADH to 10 mL prepared ALT reagent) and vortex for 15–30 s (NADH is stable in the ALT reagent only for a short period of time, i.e., approximately a couple of weeks, hence the recommendation of adding NADH to the ALT reagent right before use). If no ALT values above 250 U/L are expected, one can consider adding a lower amount of NADH, i.e., 5 mg NADH to 10 mL prepared ALT reagent.
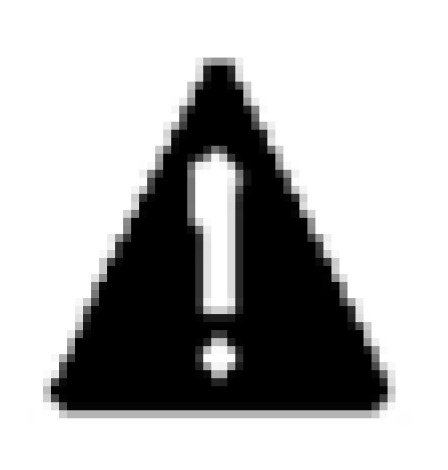
**CRITICAL STEP** Filter the required volume of ALT reagent using Whatman Puradisc 25 mm 0.2 µM PES membrane filter to remove possible remaining small particles from the solution.
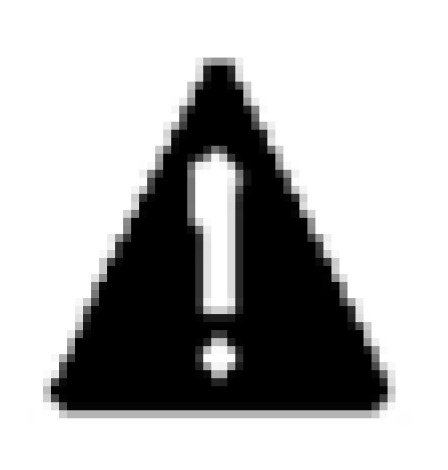
**CRITICAL STEP** Warm up the required filtered volume in a 15 mL or 50 mL Falcon tube for 5 min in a water bath maintained at 37 degrees Celsius immediately prior to pipetting to induce the kinetic reaction required for the assay to work appropriately.

### 3.3. Preparation of 96-Well Plate and ALT Measurement

15. Add 10 µL of ALT standards ranging from 0 (=water) to 1000 units/L (Validate GC3 ALT Linearity Test Kit) and plasma samples in duplicates or triplicates into 96-well flat-bottom plates.
16. Add 90 µL of filtered, warmed up ALT reagent to each sample using a multi-pipet and a disposable reagent reservoir. It is good practice to prepare 1 mL in excess of the calculated required volume to ensure rapid pipetting with the multi-pipet. Shake plate slightly after completion of pipetting. Remove (large) bubbles by bursting them with a small needle.
17. Read plate without lid in VersaMax Microplate Reader with SoftMax Pro Software, or similar microplate reader and software. 
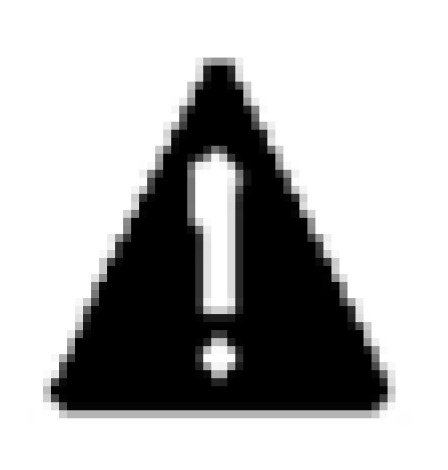
**CRITICAL STEP** Use the kinetic enzyme assay function with 24 reads at 340 nm every 13 s for a total of ~5 min. The wavelength of 340 nm is chosen to quantitate NADH, as the reduced form (NADH) can absorb light at 340 nm, whereas the oxidized from (NAD^+^) does not [17]. The more enzyme ALT is present, the more pyruvate is produced, which then in the subsequent reaction is metabolized with NADH to L-lactate and NAD+ in a reaction catalyzed by LDH. The decrease of NADH over time is detected by the kinetic assay at 340 nm and correlates with the activity of ALT [18]. The maximum reaction velocity *V_max_* is displayed in milliunits/min.

## 4. Expected Results and Discussion

### 4.1. The Stability of the Standard Curve Depends on the NADH Concentration in the ALT Reagents

**Figure 1:**
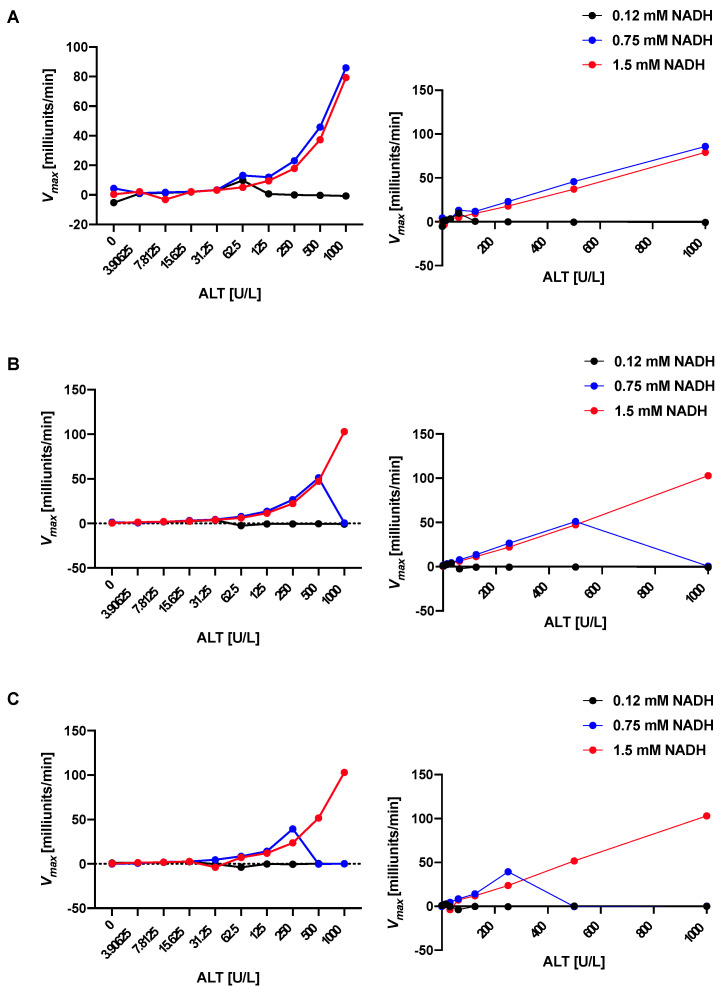
Higher concentration of reduced nicotinamide adenine dinucleotide (NADH) in alanine aminotransferase (ALT) reagent confers stability of standard curve. ALT reagent with low NADH concentration (0.12 mM, black), intermediate NADH concentration (0.75 mM, blue), and high NADH concentration (1.5 mM, red). (**A**) First run. Left, curve with Log 2 scale on *X*-axis allowing detailed view of lower ALT values; right, linear scale on *X*-axis demonstrating linear correlation of values with high NADH ALT reagent. (**B**) Second run. Left, curve with Log 2 scale on *X*-axis allowing detailed view of lower ALT values; right, linear scale on *X*-axis demonstrating linear correlation of values with high NADH ALT reagent. (**C**) Third run. Left, curve with Log 2 scale on *X*-axis allowing detailed view of lower ALT values; right, linear scale on *X*-axis demonstrating linear correlation of values with high NADH ALT reagent. *V_max_*, maximum reaction velocity.

The dependance of the standard curve on the NADH content was examined. The stability and accuracy depend on the NADH concentration with a higher NADH content (1.5 mM, red in Figure 1A–C) resulting in markedly better stability and accuracy than lower concentrations (0.12 mM, black; 0.75 mM, blue in Figure 1A–C). Table 1 shows that most of the correlation coefficients are 0.997 or higher for the high NADH content ALT reagent up to an ALT value of 1000 over the 3 subsequent runs, whereas the correlation coefficients for the ALT reagents with lower NADH concentrations are much lower. This stability over 3 runs will allow for enough time for pipetting of more complex and numerous pipetting schemes in a 96-well plate. The higher NADH concentration results in better performance and longer stability of the ALT reagent since the decrease of NADH over time is detected by the kinetic assay and correlates with the activity of ALT [18]. The ALT reagents with lower NADH concentrations are depleted sooner in the kinetic assay, resulting in inaccurate ALT values, especially in the higher ALT level range and lower reliability over time in between runs.

### 4.2. The ALT Reagent with a High NADH Content Shows Similarly Excellent Performance as a Commercial ALT and Longer Stability of the Results

**Figure 2:**
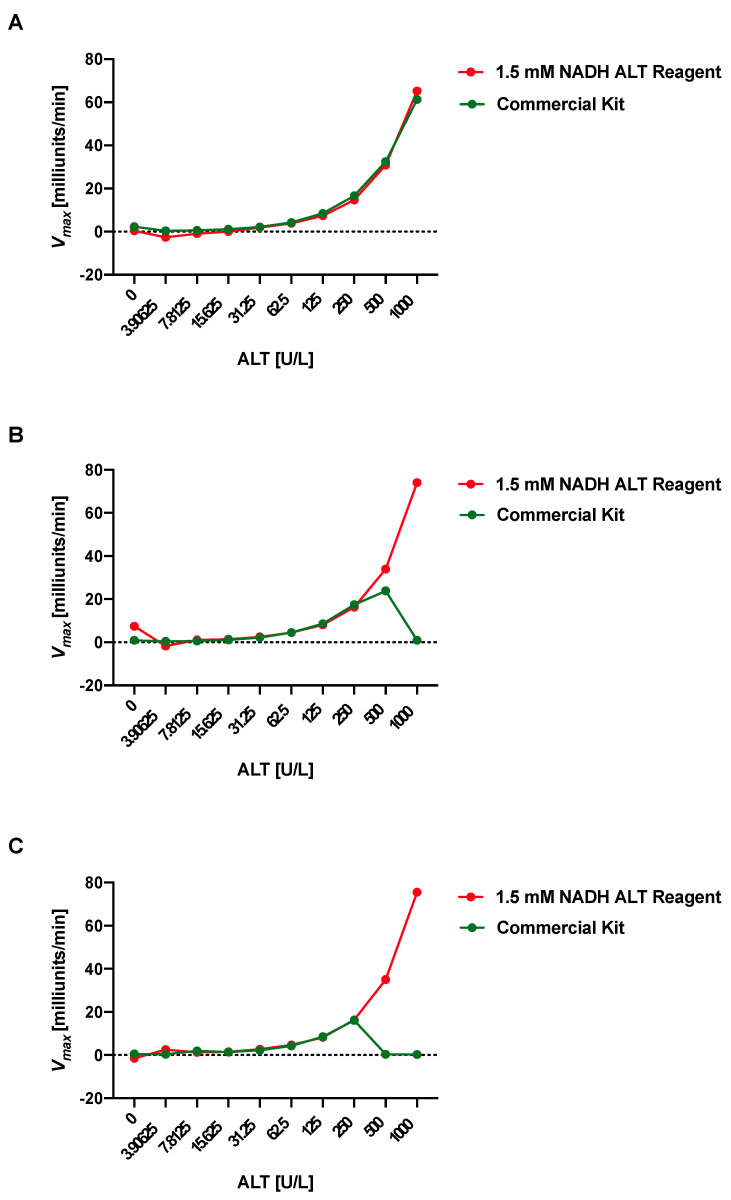
ALT reagent with high NADH concentration shows longer stability of standard curve than commercial kit. ALT reagent with high NADH concentration (1.5 mM, red) and ALT reagent from commercial kit (ALT Kinetic Assay, #A524-150, Teco Diagnostics, green). (**A**) First run. (**B**) Second run. (**C**) Third run. *V_max_*, maximum reaction velocity.

To validate the prior results, we performed a head-to-head comparison of the high NADH ALT reagent (1.5 mM, red in Figure 2A–C)–which we have used in all of the remaining experiments of the manuscript–versus a commercial kit (ALT Kinetic Assay, #A524-150, Teco Diagnostics, green in Figure 2A–C). Both ALT reagents demonstrate outstanding performance in the first run (see Table 2). However, the performance of the commercial kit diminishes during run 2 and 3 (Figure 2B,C and Table 2). The high NADH ALT reagent validates its prior excellent correlation coefficients also in run 2 and 3 (Table 2). The correlation coefficients for run 1, 2, and 3 using the high NADH ALT reagent and following the described protocol remained excellent for at least 5 months after initial preparation and storage at 4 degrees Celsius (Table 3). The high NADH ALT reagent shows high intra-run precision with a coefficient of variation (CV) of 3.59 for one sample (sample 1, *n* = 20, mean = 395.67, standard deviation [SD] = 14.22, see Supplemental Material S1 for raw data) and CV of 4.92 for another sample (sample 2, *n* = 20, mean = 197.11, SD = 9.69), and high total precision over three runs with a CV 3.65 for sample 1 (*n* = 20, mean = 400.00, SD = 14.47) and CV of 4.83 for sample 2 (*n* = 20, mean = 199.19, SD = 9.62), consistent with recommended performance per Clinical and Laboratory Standards Institute (CLSI) [19]. To determine the accuracy of the high NADH ALT reagent, the values of the first run between the high NADH ALT reagent and the commercial kit were compared and correlated with an excellent correlation coefficient of 0.998. As shown in Figure 1A–C, the assay with the high NADH ALT reagent is linear up to 1000 U/L.

### 4.3. Anticoagulation with EDTA or Heparin Results in Lower ALT Levels Compared to No Anticoagulation

**Figure 3:**
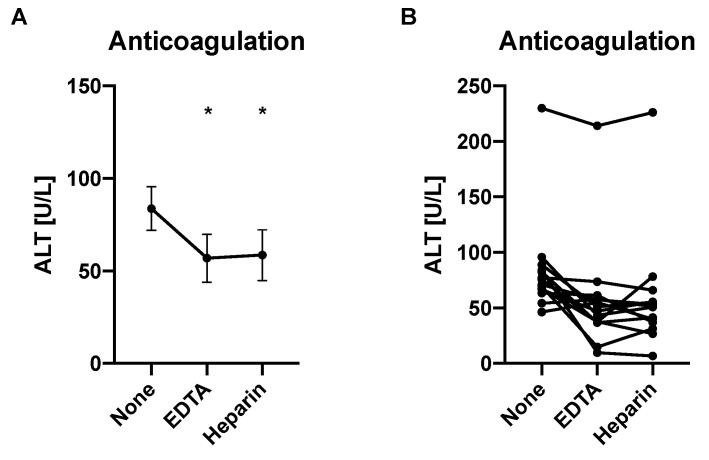
Anticoagulation with ethylenediaminetetraacetic acid (EDTA) or heparin results in lower ALT levels compared with no anticoagulation. Blood samples from mice (*n* = 14) were obtained without anticoagulation (“serum”), with EDTA (“EDTA-treated plasma”), or with heparin (“heparinized plasma”), respectively, from each mouse. (**A**) Average ALT levels for each group. (**B**) Separate ALT levels for each mouse by anticoagulation group. Results expressed as mean ± s.e.m. *p* values are determined by one-way paired ANOVA with Holm-Šídák’s post hoc test. * *p* < 0.05.

We then compared the impact of anticoagulation on ALT values. Anticoagulation with 5 µL EDTA or 10 units heparin per Eppendorf tube results in significantly lower ALT levels compared with samples without anticoagulation (Figure 3A,B). This is consistent with a prior study, in which EDTA-treated and heparinized plasma samples were found to have significantly decreased ALT levels in relation to not anticoagulated sera [20]. A possible chelating effect of EDTA was thought to contribute to this finding [20,21]. This indicates that samples of a specific set should either be all anticoagulated with EDTA or heparin (“plasma”), or not anticoagulated (“serum”) to allow for reliable comparison.

### 4.4. Hemolysis Is Associated with Elevated ALT Levels

**Figure 4:**
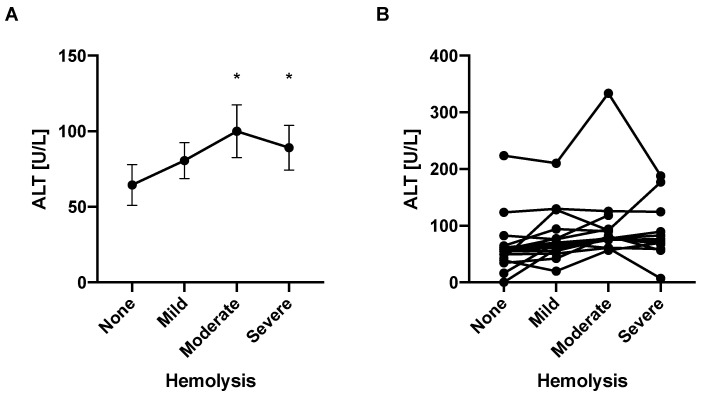
Higher degree of hemolysis is associated with higher ALT levels. Blood samples from mice (*n* = 15) were obtained with EDTA (“EDTA-treated plasma”). Hemolysis was induced for a portion of each volume by use of a microbeads beater and groups of varying degrees of hemolysis were created by mixing volumes of non-hemolyzed and hemolyzed amounts of each sample, as described in Section 2.3.2 Blood samples. The maximum reaction velocity *V_max_* values of 3 samples of the “severe hemolysis group” could not be determined by the plate reader due to marked hemolysis. (**A**) Average ALT levels for each group. (**B**) Separate ALT levels for each mouse by the degree of hemolysis. Results expressed as mean ± s.e.m. *p* values are determined by mixed-effects analysis due to missing values, and Holm-Šídák’s post hoc test with the “no hemolysis group” as the control group. * *p* < 0.05.

EDTA-treated plasma samples from a respective mouse were prepared to achieve different degrees of hemolysis (see Section 2.3.2 Blood samples). Samples with only mild hemolysis were not significantly different from non-hemolyzed samples with regard to their ALT values; however, moderately and severely hemolyzed samples demonstrated significantly higher ALT levels than non-hemolyzed samples (Figure 4A,B). After assigning values 0, 1, 2, and 3 to none, mild, moderate, and severe hemolysis groups, respectively, and testing for linear trend as part of the mixed-effects analysis using the ALT as response and numerical hemolysis as predictor, the slope estimate is 9.494 (95% confidence interval [3.040, 15.95]) with a *p* value of 0.005, indicating there is a significant correlation between a higher grade of hemolysis and higher ALT levels. This is consistent with a prior study, where clinically meaningful increases in ALT enzyme activities were observed in severely hemolyzed samples [22]. This might be due to the fact that hemoglobin absorbs strongly at 340 nm [23] and might hence lead to interference with the assay.

### 4.5. ALT Values Remain Stable over a Broad Range of Triglyceride Concentrations

**Figure 5:**
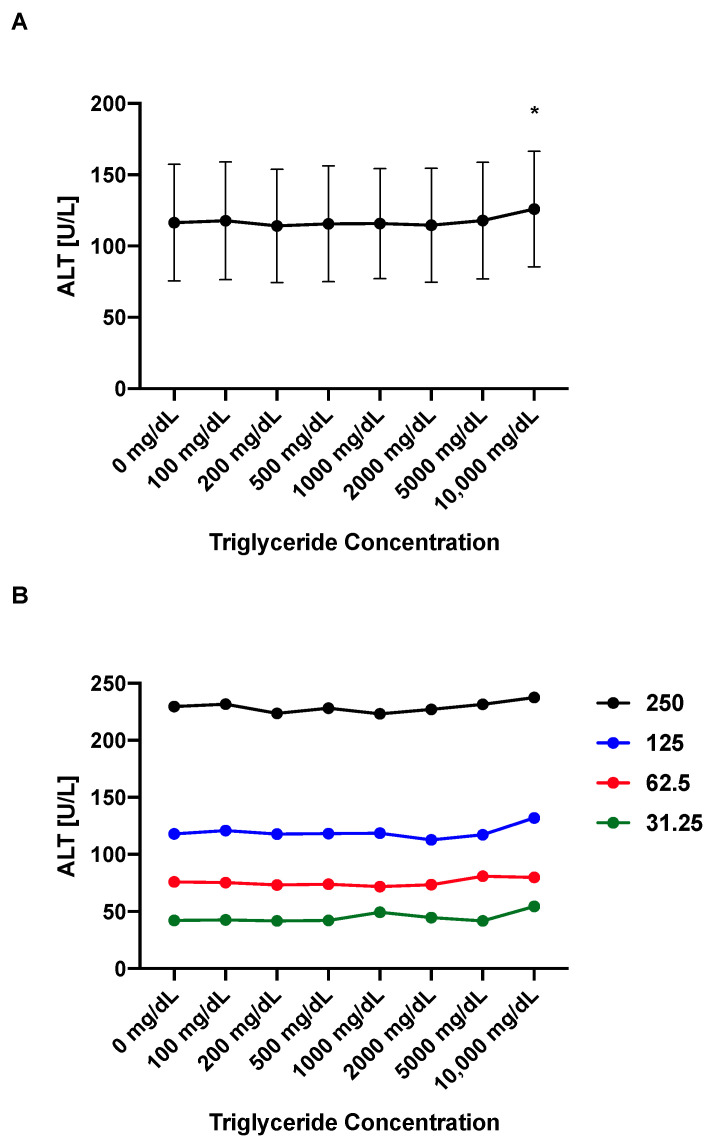
ALT values remain stable over a wide range of triglyceride concentrations. Purified triglycerides mixed with sterile water were added to respective ALT standards to achieve final ALT levels. (**A**) Mean ALT levels of ALT standards and triglyceride concentration. (**B**) Separate ALT levels for each ALT standard (250 in black, 125 in blue, 62.5 in red, and 31.25 in green) and triglyceride concentration. Results expressed as mean ± s.e.m. *p* values are determined by one-way paired ANOVA and Holm-Šídák’s post hoc test with the 0 mg/dL triglyceride group as the control group. * *p* < 0.05.

Since hyperlipidemia and hypertriglyceridemia often co-exist with elevated transaminase levels in particular in non-alcoholic fatty liver disease [5], we investigated the impact of high triglyceride concentrations on ALT levels using our assay (Figure 5A,B). The ALT levels remain stable over a wide range of triglyceride concentrations–in particular, in a biologically relevant range of up to ~1000 mg/dL triglycerides (Figure 5A) despite increasing turbidity with increasing triglyceride concentrations in the samples. The only significantly different triglyceride level compared with 0 mg/dL is 10,000 mg/dL with significantly higher ALT levels; however, this triglyceride concentration is very unlikely to be present in rodents or humans outside of rare familial syndromes [24]. The high NADH ALT reagent shows high precision across a wide triglyceride range of 0–10,000 mg/dL with a CV of 10.35 for ALT standard 31.25 (*n* = 8 for all standards, see Supplemental Material S1 for raw data), CV of 4.35 for ALT standard 62.5, CV of 4.62 for ALT standard 125, and CV of 2.04 for ALT standard 250; and across a wide biological more relevant triglyceride range 0–500 mg/dL with a CV of 0.92 for ALT standard 31.25, CV of 1.70 for ALT standard 62.5, CV of 1.24 for ALT standard 125, and CV of 1.51 for ALT standard 250. Our results with a high stability over a wide range of triglyceride concentrations are in line with results from commercial ALT kits [25].

### 4.6. ALT Levels Decrease in Diluted Samples Proportionally to the Dilution Factor

**Figure 6:**
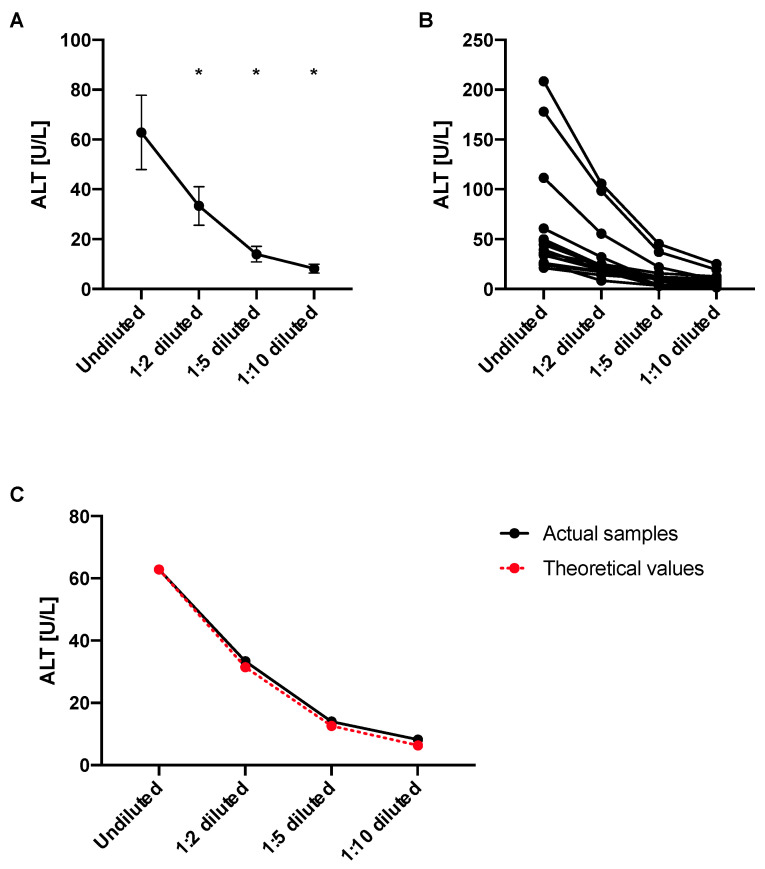
Decrease of ALT values of blood samples follows their dilution factor. Blood samples from mice (*n* = 15) were obtained with EDTA (“plasma”) and diluted as indicated. (**A**) Average ALT levels for each group. (**B**) Separate ALT levels for each mouse by dilution factor. (**C**) Average ALT levels of actual samples in black compared with theoretical values in red. Results expressed as mean ± s.e.m. *p* values are determined by one-way paired ANOVA with Holm-Šídák’s post hoc test. * *p* < 0.05.

Next, we wanted to investigate whether ALT levels are still reliable in diluted plasma samples employing our ALT assay. We found that the ALT values diminish according to their dilution factor, i.e., one would expect a similar decrease in ALT value in the same magnitude of the factor that the samples were diluted with. No significant differences were noted between actual values and theoretical values (Figure 6A–C). Although not significant, the theoretical values were lower by 6.20%, 11.38%, and 30.00% than the actual values for 1:2, 1:5, and 1:10 dilution, respectively. We would hence still recommend to only compare samples of the same dilution.

### 4.7. The Presented ALT Assay Is Less Expensive Than Commercial ALT Kits

As described above, in order to prepare 100 mL ALT reagent, one requires 1.175 g Tris Base (1 kg, #BP152-1, Fisher Scientific, $252), 190.0 mg alpha-ketoglutarate (25 g, #75890-25G, Sigma Aldrich, $35.62), 3.92 g L-alanine (100 g, #A7627-100G, Sigma Aldrich, $60.76), 1 mL of 0.5 M EDTA (500 mL, #E177-500 ML, VWR, $66.19), 100 mg NADH (1 g, #16078, Cayman Chemical, $52), and 320 units of LDH (1000 units, #350-20-1, Lee Bio, $27). This corresponds to $17.28 (=$0.30 + $0.27 + $2.38 + $0.13 + $5.20 + ~$9.00). Using 90 µL ALT reagent per sample, 100 mL would allow 1111 reactions. The final cost for the described assay would be approximately 1.56 cents per sample. On the contrary, a representative commercial assay used in this manuscript for comparison (ALT Kinetic Assay, #A524-150, Teco Diagnostics) costs $200 and allows for 150 reactions if used per manufacturer’s instructions, corresponding to $1.33 per reaction. This demonstrates how cost-effective the described ALT assay is compared with commercial assays.

## 5. Conclusions

In conclusion, here we present an inexpensive, accurate, reliable, and scalable methodology to quantify liver cell injury. This can be applied to a broad spectrum of liver diseases in mice and possibly humans.

## Figures and Tables

**Table 1:** Correlation coefficients between ALT [U/L] and maximum reaction velocity [milliunits/min] depend on NADH concentration in ALT reagent.

	0.12 mM NADH	0.75 mM NADH	1.5 mM NADH
First Run			
0 to 1000	−0.219	0.996	0.998
7.8125 to 1000	−0.489	0.997	0.998
0 to 500	−0.138	0.987	0.992
Second Run			
0 to 1000	−0.427	0.274	0.998
7.8125 to 1000	−0.405	0.188	0.998
0 to 500	−0.438	1.000	0.999
Third Run			
0 to 1000	−0.112	−0.054	0.997
7.8125 to 1000	−0.024	−0.166	0.997
0 to 500	−0.148	0.253	0.989

**Table 2:** Correlation coefficients between ALT [U/L] and maximum reaction velocity [milliunits/min] in comparison with commercial Kit.

	1.5 mM NADH	Commercial Kit
First Run		
0 to 1000	0.999	0.999
7.8125 to 1000	1.000	1.000
0 to 500	0.996	0.998
Second Run		
0 to 1000	0.993	0.276
7.8125 to 1000	0.999	0.185
0 to 500	0.972	0.979
Third Run		
0 to 1000	0.998	−0.085
7.8125 to 1000	0.999	−0.212
0 to 500	0.996	0.240

**Table 3:** Correlation coefficients between ALT [U/L] and maximum reaction velocity [milliunits/min] are stable for the high NADH ALT reagent for at least 5 months.

	1 Month	2 Months	5 Months
First Run			
0 to 1000	0.993	0.980	0.990
7.8125 to 1000	0.998	0.997	0.991
0 to 500	0.970	0.918	0.969
Second Run			
0 to 1000	0.999	0.998	0.999
7.8125 to 1000	1.000	0.998	1.000
0 to 500	0.997	0.991	0.996
Third Run			
0 to 1000	0.999	0.997	0.997
7.8125 to 1000	1.000	0.999	0.997
0 to 500	0.997	0.992	0.989

## Data Availability

The raw data is included in Supplementary S1.

## Supplementary Materials

The following supporting information can be downloaded at: https://www.mdpi.com/article/10.3390/mps5050081/s1, Supplemental Material S1: Raw Data.
